# (1*R*,3*S*,5*R*,6*S*)-6-Hydr­oxy-3-tosyl­oxy­tropan-8-ium chloride

**DOI:** 10.1107/S1600536809055366

**Published:** 2010-01-09

**Authors:** Nian-Xi Yu, Li-Min Yang, Yang Lu

**Affiliations:** a Department of Pharmacy, Shanghai Jiao Tong University School of Medicine, South Chongqing Road 280, Shanghai 200025, People’s Republic of China

## Abstract

The title compound, C_15_H_22_NO_4_S^+^·Cl^−^, is a hydrolysis product of lesatropane [(1*R*,3*S*,5*R*,6*S*)-6-acet­oxy-3-tosyl­oxytropane] hydro­chloride, a potential anti­glaucoma agent. As in lesatropane, the piperidine and pyrrolidine rings in the title compound adopt chair and envelope conformations, respectively. There are two mol­ecules in the unit cell with similar conformations. The crystal structure is stabilized by inter­molecular O—H⋯Cl and N—H⋯Cl hydrogen bonds.

## Related literature

For background to the pharmacological activity of lesatropane, see: Zhu *et al.* (2008 ▶); Fu *et al.* (2008 ▶, 2009 ▶). For related structures, see: Yang *et al.* (2008 ▶, 2009 ▶).
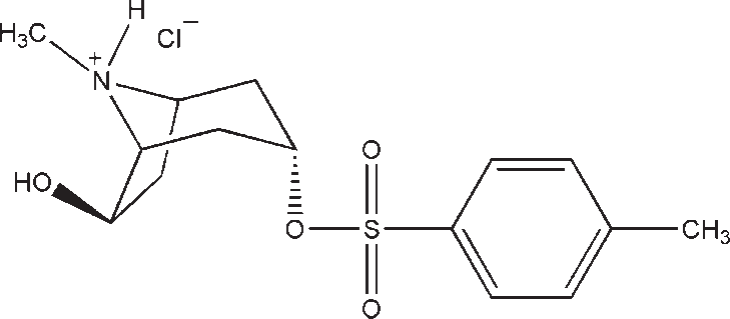


## Experimental

### 

#### Crystal data

C_15_H_22_NO_4_S^+^·Cl^−^
*M* *_r_* = 347.85
Triclinic, 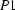

*a* = 7.1958 (9) Å
*b* = 9.3680 (12) Å
*c* = 13.4124 (17) Å
α = 69.894 (2)°
β = 76.790 (2)°
γ = 85.560 (2)°
*V* = 826.57 (18) Å^3^
*Z* = 2
Mo *K*α radiation
μ = 0.37 mm^−1^
*T* = 293 K
0.39 × 0.28 × 0.15 mm

#### Data collection

Bruker SMART CCD area-detector diffractometer
Absorption correction: multi-scan (*SADABS*; Sheldrick, 2002 ▶) *T*_min_ = 0.754, *T*_max_ = 1.000
4886 measured reflections
4168 independent reflections
3497 reflections with *I* > 2σ(*I*)
*R*_int_ = 0.014

#### Refinement

*R*[*F* ^2^ > 2σ(*F* ^2^)] = 0.043
*wR*(*F* ^2^) = 0.106
*S* = 0.98
4168 reflections
417 parameters
7 restraints
H atoms treated by a mixture of independent and constrained refinement
Δρ_max_ = 0.37 e Å^−3^
Δρ_min_ = −0.24 e Å^−3^
Absolute structure: Flack (1983 ▶), 3603 Friedel pairs
Flack parameter: −0.15 (8)

### 

Data collection: *SMART* (Bruker, 2001 ▶); cell refinement: *SAINT* (Bruker, 2001 ▶); data reduction: *SAINT*; program(s) used to solve structure: *SHELXS97* (Sheldrick, 2008 ▶); program(s) used to refine structure: *SHELXL97* (Sheldrick, 2008 ▶); molecular graphics: *SHELXTL* (Sheldrick, 2008 ▶); software used to prepare material for publication: *SHELXTL*.

## Supplementary Material

Crystal structure: contains datablocks I, global. DOI: 10.1107/S1600536809055366/bq2182sup1.cif

Structure factors: contains datablocks I. DOI: 10.1107/S1600536809055366/bq2182Isup2.hkl

Additional supplementary materials:  crystallographic information; 3D view; checkCIF report

## Figures and Tables

**Table 1:** Hydrogen-bond geometry (Å, °)

*D*—H⋯*A*	*D*—H	H⋯*A*	*D*⋯*A*	*D*—H⋯*A*
O1—H1*A*⋯Cl1^i^	0.82 (2)	2.37 (2)	3.185 (4)	170 (6)
O5—H5⋯Cl2^ii^	0.83 (2)	2.35 (3)	3.156 (4)	164 (6)
N1—H1*B*⋯Cl1	0.87 (2)	2.26 (2)	3.091 (4)	159 (4)
N2—H2⋯Cl2	0.87 (2)	2.19 (2)	3.041 (4)	165 (4)

Symmetry codes: (i) 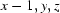; (ii) 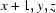.

**Fig. 1.**
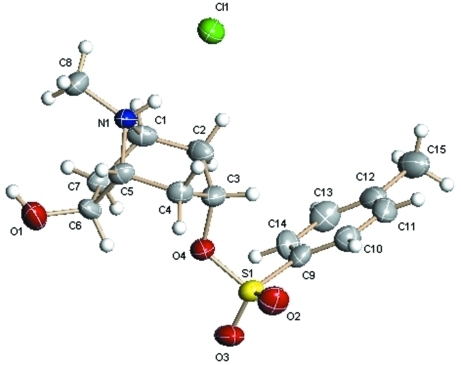
The molecular structure of the title compound with displacement ellipsoids drawn at the 50% probability level.

**Fig. 2.**
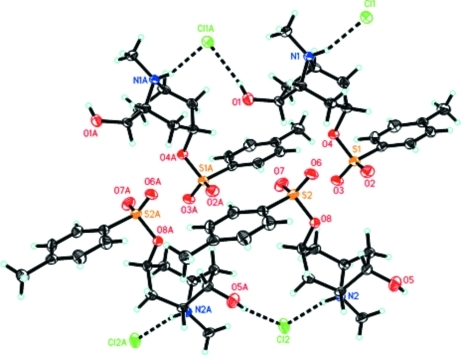
The intermolecular O—H···Cl and N—H···Cl hydrogen bonds (dashed lines). Symmetry codes: (Cl 1 A) x - 1, y, z; (Cl 2 A) x + 1, y, z.

## supplementary crystallographic information

### Comment

Lesatropane has been demonstrated to possess potent agonistic activity on muscarinic receptors (Zhu *et al., *2008) and is being developed into a new antiglaucoma agent in China. The ocular pharmacokinetics of lesatropane was evaluated (Fu *et al.*, 2008, 2009). The related crystal structures have been reported (Yang *et al.*, 2008, 2009). The title compound was detected during storage of lesatropane hydrochloride solution. We prepared the title compound (Fig.1), and report here its crystal structure. The piperidine ring exists in a chair conformation with N1 atom and C3 atom displaced by 0.881 (6) Å and -0.446 (7) Å, respectively, on opposite sides of C1/C2/C4/C5 plane. The pyrrolidine ring adopts an envelope conformation with N1 atom deviating by 0.695 (7)Å from C1/C5/C6/C7 plane. There are two molecules in the unit cell, and the dihedral angle between two benzene rings is 1.65 (34)°. The molecules are linked to each other by intermolecular strong O—H···Cl and N—H···Cl hydrogen bonds. (Table 1. and Fig. 2.).

### Experimental

Lesatropane was hydrolyzed with 1% NaOH-EtOH solution. After stirring at 0°C for 5 h, the reaction solution was neutralized by hydrochloric acid. The solvent was evaporated *in vacuo*. The residue was dissolved in CH_2_Cl_2_ and the organic phase was evaporated *in vacuo* to give the title compound as colorless crystals, [α]_D_^25^-8.31(c= 0.1085,CHCl_3_).^1^H NMR(CDCl_3_): δ1.98–3.00(m, 6H, 2,4,7-H), 2.47(s, 3H, CH_3_Ar), 3.01(s, 3H, CH_3_N), 3.80(s, 1H, 1-H), 3.87(m, 1H, 5-H), 4.19(s, 1H, OH), 4.63(t, 1H, 3-H), 4.94(m, 1H, 6-H), 7.38(d, J=8.1 Hz, 2H, ArH), 7.75(d, J=8.1 Hz, 2H, ArH), 11.75(s, 1H, H^+^). Crystals suitable for X-ray analysis were obtained by slow crystallization from anhydrous ethanol.

### Refinement

H atoms bonded to N atom was located in a difference map and refined with distance restraints of N—H=0.873 (19) Å, other H atoms were positioned geometrically and allowed to ride on their parent atoms. with C—H=0.93–0.98 Å, O—H=0.83 (2) Å,. Displacement parameters for H atoms were calculated as *U*_iso_(H) = 1.2–1.5Ueq(carrier atom).

The title compound is a chiral compound and although 95% symmetry center was detected which means that P1 should be P-1, P-1 is in contradiction with the chirality. P-1 was also tried to refine the structure but gave R=0.084. So, P1 is more reasonable and credible.

### Crystal data

**Table:**

C_15_H_22_NO_4_S^+^·Cl^−^	*Z* = 2
*M**_r_* = 347.85	*F*(000) = 368
Triclinic, *P*1	*D*_x_ = 1.398 Mg m^−^^3^
Hall symbol: P 1	Melting point: 412 K
*a* = 7.1958 (9) Å	Mo *K*α radiation, λ = 0.71073 Å
*b* = 9.3680 (12) Å	Cell parameters from 2053 reflections
*c* = 13.4124 (17) Å	θ = 4.6–54.7°
α = 69.894 (2)°	µ = 0.37 mm^−^^1^
β = 76.790 (2)°	*T* = 293 K
γ = 85.560 (2)°	Prismatic, colorless
*V* = 826.57 (18) Å^3^	0.39 × 0.28 × 0.15 mm

### Data collection

**Table:**

Bruker SMART CCD area-detector diffractometer	4168 independent reflections
Radiation source: fine-focus sealed tube	3497 reflections with *I* > 2σ(*I*)
graphite	*R*_int_ = 0.014
phi and ω scans	θ_max_ = 27.0°, θ_min_ = 1.7°
Absorption correction: multi-scan (*SADABS*; Sheldrick, 2002)	*h* = −8→9
*T*_min_ = 0.754, *T*_max_ = 1.000	*k* = −11→11
4886 measured reflections	*l* = −17→17

### Refinement

**Table:**

Refinement on *F*^2^	Secondary atom site location: difference Fourier map
Least-squares matrix: full	Hydrogen site location: inferred from neighbouring sites
*R*[*F*^2^ > 2σ(*F*^2^)] = 0.043	H atoms treated by a mixture of independent and constrained refinement
*wR*(*F*^2^) = 0.106	*w* = 1/[σ^2^(*F*_o_^2^) + (0.060*P*)^2^ + ] where *P* = (*F*_o_^2^ + 2*F*_c_^2^)/3
*S* = 0.98	(Δ/σ)_max_ = 0.001
4168 reflections	Δρ_max_ = 0.37 e Å^−^^3^
417 parameters	Δρ_min_ = −0.24 e Å^−^^3^
7 restraints	Absolute structure: Flack (1983), 3603 Friedel pairs
Primary atom site location: structure-invariant direct methods	Flack parameter: −0.15 (8)

### Special details

**Table:**

Geometry. All e.s.d.'s (except the e.s.d. in the dihedral angle between two l.s. planes) are estimated using the full covariance matrix. The cell e.s.d.'s are taken into account individually in the estimation of e.s.d.'s in distances, angles and torsion angles; correlations between e.s.d.'s in cell parameters are only used when they are defined by crystal symmetry. An approximate (isotropic) treatment of cell e.s.d.'s is used for estimating e.s.d.'s involving l.s. planes.
Refinement. Refinement of *F*^2^ against ALL reflections. The weighted *R*-factor *wR* and goodness of fit *S* are based on *F*^2^, conventional *R*-factors *R* are based on *F*, with *F* set to zero for negative *F*^2^. The threshold expression of *F*^2^ > σ(*F*^2^) is used only for calculating *R*-factors(gt) *etc*. and is not relevant to the choice of reflections for refinement. *R*-factors based on *F*^2^ are statistically about twice as large as those based on *F*, and *R*- factors based on ALL data will be even larger.

### Fractional atomic coordinates and isotropic or equivalent isotropic displacement parameters (Å^2^)

**Table:**

	*x*	*y*	*z*	*U*_iso_*/*U*_eq_	
S1	0.68669 (16)	0.49958 (12)	0.53438 (8)	0.0397 (3)	
S2	0.41766 (16)	0.19107 (12)	0.44619 (8)	0.0401 (3)	
Cl1	0.73995 (19)	0.03363 (15)	1.03456 (10)	0.0487 (3)	
Cl2	0.35173 (19)	0.66678 (16)	−0.03841 (11)	0.0622 (4)	
O1	0.0387 (5)	0.1347 (4)	0.8073 (3)	0.0619 (9)	
O2	0.8064 (6)	0.3982 (5)	0.4922 (3)	0.0541 (10)	
O3	0.5651 (6)	0.5988 (4)	0.4705 (3)	0.0548 (10)	
O4	0.5462 (5)	0.4084 (4)	0.6436 (3)	0.0418 (8)	
O5	1.0882 (5)	0.3794 (4)	0.0936 (3)	0.0576 (8)	
O6	0.5406 (6)	0.0955 (4)	0.5103 (3)	0.0527 (10)	
O7	0.2965 (6)	0.2969 (5)	0.4846 (3)	0.0591 (11)	
O8	0.5575 (4)	0.2796 (4)	0.3366 (2)	0.0376 (8)	
N1	0.3883 (6)	0.1187 (5)	0.9298 (3)	0.0359 (9)	
N2	0.7097 (6)	0.5851 (4)	0.0538 (3)	0.0354 (9)	
C1	0.3815 (8)	0.2874 (6)	0.8983 (4)	0.0427 (13)	
H1	0.3514	0.3175	0.9633	0.051*	
C2	0.5693 (8)	0.3497 (6)	0.8305 (4)	0.0443 (13)	
H2A	0.6643	0.3172	0.8747	0.053*	
H2B	0.5632	0.4598	0.8072	0.053*	
C3	0.6333 (8)	0.3018 (6)	0.7310 (4)	0.0392 (11)	
H3	0.7726	0.3097	0.7079	0.047*	
C4	0.5728 (7)	0.1407 (5)	0.7498 (4)	0.0375 (11)	
H4A	0.6666	0.0698	0.7810	0.045*	
H4B	0.5712	0.1310	0.6803	0.045*	
C5	0.3765 (7)	0.0975 (5)	0.8249 (3)	0.0355 (11)	
H5A	0.3451	−0.0080	0.8375	0.043*	
C6	0.2166 (7)	0.2045 (6)	0.7864 (4)	0.0411 (12)	
H6	0.2568	0.2542	0.7076	0.049*	
C7	0.2132 (8)	0.3253 (6)	0.8403 (4)	0.0448 (13)	
H7A	0.0934	0.3214	0.8921	0.054*	
H7B	0.2283	0.4261	0.7860	0.054*	
C8	0.2418 (8)	0.0359 (7)	1.0276 (4)	0.0503 (13)	
H8A	0.2770	0.0380	1.0919	0.075*	
H8B	0.2342	−0.0677	1.0311	0.075*	
H8C	0.1199	0.0839	1.0226	0.075*	
C9	0.8273 (8)	0.6055 (5)	0.5760 (4)	0.0370 (11)	
C10	1.0154 (8)	0.5730 (6)	0.5736 (4)	0.0442 (13)	
H10	1.0718	0.4957	0.5483	0.053*	
C11	1.1224 (8)	0.6543 (6)	0.6087 (4)	0.0457 (13)	
H11	1.2519	0.6337	0.6049	0.055*	
C12	1.0377 (9)	0.7672 (6)	0.6497 (4)	0.0476 (14)	
C13	0.8473 (10)	0.7977 (7)	0.6502 (5)	0.0538 (16)	
H13	0.7891	0.8746	0.6754	0.065*	
C14	0.7414 (8)	0.7177 (6)	0.6147 (4)	0.0466 (13)	
H14	0.6125	0.7390	0.6167	0.056*	
C15	1.1547 (10)	0.8524 (7)	0.6924 (5)	0.0634 (16)	
H15A	1.2123	0.7809	0.7475	0.095*	
H15B	1.0731	0.9197	0.7230	0.095*	
H15C	1.2527	0.9102	0.6338	0.095*	
C16	0.6975 (7)	0.6088 (5)	0.1618 (4)	0.0365 (11)	
H16	0.7089	0.7166	0.1520	0.044*	
C17	0.5067 (8)	0.5446 (6)	0.2326 (4)	0.0417 (12)	
H17A	0.4967	0.5536	0.3035	0.050*	
H17B	0.4067	0.6066	0.2009	0.050*	
C18	0.4711 (7)	0.3819 (5)	0.2478 (4)	0.0370 (11)	
H18	0.3332	0.3637	0.2669	0.044*	
C19	0.5583 (8)	0.3388 (6)	0.1467 (4)	0.0395 (12)	
H19A	0.5768	0.2296	0.1687	0.047*	
H19B	0.4688	0.3651	0.0990	0.047*	
C20	0.7488 (7)	0.4169 (5)	0.0834 (4)	0.0337 (10)	
H20	0.7966	0.3892	0.0180	0.040*	
C21	0.9008 (7)	0.3873 (6)	0.1520 (4)	0.0400 (12)	
H21	0.8709	0.2909	0.2120	0.048*	
C22	0.8696 (8)	0.5180 (6)	0.1996 (4)	0.0428 (12)	
H22A	0.8450	0.4775	0.2784	0.051*	
H22B	0.9819	0.5824	0.1735	0.051*	
C23	0.8482 (8)	0.6850 (6)	−0.0388 (4)	0.0468 (13)	
H23A	0.8071	0.7887	−0.0534	0.070*	
H23B	0.8558	0.6567	−0.1019	0.070*	
H23C	0.9716	0.6743	−0.0210	0.070*	
C24	0.2817 (7)	0.0842 (6)	0.4053 (4)	0.0353 (11)	
C25	0.0900 (8)	0.1097 (6)	0.4105 (4)	0.0432 (13)	
H25	0.0277	0.1837	0.4377	0.052*	
C26	−0.0087 (8)	0.0230 (7)	0.3746 (4)	0.0513 (14)	
H26	−0.1392	0.0389	0.3797	0.062*	
C27	0.0761 (9)	−0.0845 (6)	0.3321 (4)	0.0449 (13)	
C28	0.2681 (9)	−0.1099 (7)	0.3296 (5)	0.0546 (16)	
H28	0.3289	−0.1844	0.3025	0.066*	
C29	0.3729 (8)	−0.0288 (7)	0.3658 (5)	0.0511 (15)	
H29	0.5021	−0.0489	0.3640	0.061*	
C30	−0.0316 (10)	−0.1748 (8)	0.2911 (5)	0.077 (2)	
H30A	−0.1536	−0.2033	0.3396	0.116*	
H30B	0.0393	−0.2646	0.2873	0.116*	
H30C	−0.0498	−0.1144	0.2199	0.116*	
H1A	−0.030 (7)	0.116 (6)	0.868 (2)	0.09 (2)*	
H5	1.137 (8)	0.463 (4)	0.056 (4)	0.09 (2)*	
H1B	0.503 (4)	0.097 (5)	0.942 (4)	0.034 (13)*	
H2	0.600 (4)	0.616 (5)	0.037 (4)	0.036 (14)*	

### Atomic displacement parameters (Å^2^)

**Table:**

	*U*^11^	*U*^22^	*U*^33^	*U*^12^	*U*^13^	*U*^23^
S1	0.0434 (8)	0.0448 (8)	0.0283 (6)	−0.0043 (6)	−0.0064 (6)	−0.0088 (6)
S2	0.0463 (8)	0.0432 (7)	0.0303 (6)	−0.0095 (6)	−0.0023 (6)	−0.0135 (6)
Cl1	0.0452 (8)	0.0527 (7)	0.0471 (7)	0.0030 (6)	−0.0155 (6)	−0.0124 (6)
Cl2	0.0449 (9)	0.0580 (9)	0.0738 (10)	−0.0037 (7)	−0.0283 (8)	0.0012 (8)
O1	0.043 (2)	0.098 (3)	0.052 (2)	−0.0107 (18)	−0.0097 (17)	−0.032 (2)
O2	0.062 (3)	0.059 (2)	0.049 (2)	0.001 (2)	−0.0074 (19)	−0.031 (2)
O3	0.062 (3)	0.063 (3)	0.0350 (19)	−0.008 (2)	−0.021 (2)	−0.0025 (19)
O4	0.042 (2)	0.045 (2)	0.0321 (17)	−0.0066 (16)	−0.0102 (16)	−0.0021 (16)
O5	0.0418 (19)	0.057 (2)	0.065 (2)	0.0080 (16)	−0.0030 (16)	−0.0161 (18)
O6	0.064 (3)	0.056 (2)	0.035 (2)	−0.0118 (19)	−0.021 (2)	−0.0028 (18)
O7	0.059 (3)	0.063 (3)	0.056 (2)	−0.008 (2)	0.010 (2)	−0.033 (2)
O8	0.032 (2)	0.045 (2)	0.0309 (17)	−0.0034 (14)	−0.0077 (15)	−0.0046 (15)
N1	0.032 (2)	0.047 (3)	0.0269 (19)	0.0011 (19)	−0.0079 (18)	−0.0092 (18)
N2	0.035 (2)	0.036 (2)	0.031 (2)	−0.0019 (18)	−0.0093 (19)	−0.0029 (18)
C1	0.061 (4)	0.041 (3)	0.033 (2)	0.007 (2)	−0.019 (2)	−0.018 (2)
C2	0.055 (3)	0.039 (3)	0.042 (3)	−0.010 (2)	−0.020 (3)	−0.009 (2)
C3	0.038 (3)	0.045 (3)	0.028 (2)	0.001 (2)	−0.008 (2)	−0.005 (2)
C4	0.045 (3)	0.039 (3)	0.032 (2)	0.005 (2)	−0.009 (2)	−0.017 (2)
C5	0.047 (3)	0.033 (2)	0.027 (2)	−0.002 (2)	−0.005 (2)	−0.0111 (19)
C6	0.030 (3)	0.064 (3)	0.031 (2)	0.004 (2)	−0.011 (2)	−0.016 (2)
C7	0.042 (3)	0.056 (3)	0.036 (2)	0.013 (2)	−0.007 (2)	−0.019 (2)
C8	0.043 (3)	0.063 (3)	0.034 (3)	−0.007 (2)	−0.002 (2)	−0.004 (2)
C9	0.040 (3)	0.034 (3)	0.029 (2)	−0.007 (2)	0.000 (2)	−0.004 (2)
C10	0.045 (3)	0.048 (3)	0.041 (3)	0.002 (2)	−0.007 (3)	−0.018 (3)
C11	0.040 (3)	0.048 (3)	0.043 (3)	0.002 (2)	−0.013 (2)	−0.006 (2)
C12	0.056 (4)	0.049 (3)	0.031 (3)	−0.014 (3)	−0.010 (3)	−0.003 (2)
C13	0.064 (4)	0.043 (3)	0.056 (4)	0.001 (3)	−0.005 (3)	−0.025 (3)
C14	0.032 (3)	0.050 (3)	0.057 (3)	0.006 (2)	−0.009 (3)	−0.020 (3)
C15	0.081 (5)	0.059 (4)	0.049 (3)	−0.024 (3)	−0.019 (3)	−0.008 (3)
C16	0.040 (3)	0.034 (2)	0.039 (2)	0.004 (2)	−0.010 (2)	−0.017 (2)
C17	0.042 (3)	0.046 (3)	0.034 (2)	0.008 (2)	−0.005 (2)	−0.013 (2)
C18	0.030 (3)	0.040 (3)	0.039 (3)	0.001 (2)	−0.010 (2)	−0.009 (2)
C19	0.049 (3)	0.039 (3)	0.035 (3)	0.003 (2)	−0.015 (2)	−0.015 (2)
C20	0.038 (3)	0.039 (3)	0.025 (2)	0.0008 (19)	−0.0006 (19)	−0.015 (2)
C21	0.034 (3)	0.044 (3)	0.035 (2)	0.004 (2)	−0.008 (2)	−0.006 (2)
C22	0.047 (3)	0.049 (3)	0.035 (2)	−0.013 (2)	−0.008 (2)	−0.016 (2)
C23	0.044 (3)	0.055 (3)	0.030 (3)	0.001 (2)	−0.002 (2)	−0.004 (2)
C24	0.038 (3)	0.038 (3)	0.029 (2)	−0.002 (2)	−0.008 (2)	−0.009 (2)
C25	0.038 (3)	0.040 (3)	0.043 (3)	0.003 (2)	−0.004 (2)	−0.008 (2)
C26	0.031 (3)	0.066 (4)	0.051 (3)	−0.011 (2)	−0.008 (3)	−0.010 (3)
C27	0.057 (4)	0.044 (3)	0.029 (2)	−0.015 (2)	−0.008 (2)	−0.004 (2)
C28	0.061 (4)	0.050 (3)	0.063 (4)	0.003 (3)	−0.019 (3)	−0.029 (3)
C29	0.053 (4)	0.049 (3)	0.061 (4)	0.002 (3)	−0.014 (3)	−0.029 (3)
C30	0.087 (5)	0.098 (5)	0.053 (4)	−0.045 (4)	−0.017 (4)	−0.021 (4)

### Geometric parameters (Å, °)

**Table:**

S1—O2	1.409 (3)	C10—C11	1.377 (8)
S1—O3	1.426 (4)	C10—H10	0.9300
S1—O4	1.579 (4)	C11—C12	1.394 (7)
S1—C9	1.765 (6)	C11—H11	0.9300
S2—O6	1.421 (4)	C12—C13	1.377 (8)
S2—O7	1.421 (4)	C12—C15	1.520 (8)
S2—O8	1.573 (3)	C13—C14	1.367 (8)
S2—C24	1.741 (5)	C13—H13	0.9300
Cl1—H1B	2.26 (2)	C14—H14	0.9300
Cl2—H2	2.19 (2)	C15—H15A	0.9600
O1—C6	1.409 (6)	C15—H15B	0.9600
O1—H1A	0.82 (2)	C15—H15C	0.9600
O4—C3	1.482 (6)	C16—C17	1.512 (7)
O5—C21	1.406 (6)	C16—C22	1.530 (7)
O5—H5	0.83 (2)	C16—H16	0.9800
O8—C18	1.478 (5)	C17—C18	1.501 (7)
N1—C1	1.489 (6)	C17—H17A	0.9700
N1—C8	1.493 (7)	C17—H17B	0.9700
N1—C5	1.509 (5)	C18—C19	1.531 (6)
N1—H1B	0.874 (19)	C18—H18	0.9800
N2—C23	1.480 (6)	C19—C20	1.527 (7)
N2—C20	1.507 (6)	C19—H19A	0.9700
N2—C16	1.522 (6)	C19—H19B	0.9700
N2—H2	0.873 (19)	C20—C21	1.536 (6)
C1—C2	1.480 (7)	C20—H20	0.9800
C1—C7	1.540 (7)	C21—C22	1.544 (6)
C1—H1	0.9800	C21—H21	0.9800
C2—C3	1.514 (6)	C22—H22A	0.9700
C2—H2A	0.9700	C22—H22B	0.9700
C2—H2B	0.9700	C23—H23A	0.9600
C3—C4	1.524 (7)	C23—H23B	0.9600
C3—H3	0.9800	C23—H23C	0.9600
C4—C5	1.527 (7)	C24—C25	1.372 (7)
C4—H4A	0.9700	C24—C29	1.398 (6)
C4—H4B	0.9700	C25—C26	1.383 (8)
C5—C6	1.527 (6)	C25—H25	0.9300
C5—H5A	0.9800	C26—C27	1.361 (7)
C6—C7	1.535 (7)	C26—H26	0.9300
C6—H6	0.9800	C27—C28	1.378 (8)
C7—H7A	0.9700	C27—C30	1.495 (8)
C7—H7B	0.9700	C28—C29	1.374 (8)
C8—H8A	0.9600	C28—H28	0.9300
C8—H8B	0.9600	C29—H29	0.9300
C8—H8C	0.9600	C30—H30A	0.9600
C9—C10	1.360 (7)	C30—H30B	0.9600
C9—C14	1.372 (6)	C30—H30C	0.9600
			
O2—S1—O3	119.2 (2)	C13—C12—C15	122.0 (5)
O2—S1—O4	110.1 (2)	C11—C12—C15	120.1 (6)
O3—S1—O4	104.3 (2)	C14—C13—C12	121.7 (5)
O2—S1—C9	108.6 (2)	C14—C13—H13	119.2
O3—S1—C9	110.0 (2)	C12—C13—H13	119.2
O4—S1—C9	103.5 (2)	C13—C14—C9	119.3 (5)
O6—S2—O7	120.3 (2)	C13—C14—H14	120.3
O6—S2—O8	103.7 (2)	C9—C14—H14	120.3
O7—S2—O8	109.2 (2)	C12—C15—H15A	109.5
O6—S2—C24	110.6 (2)	C12—C15—H15B	109.5
O7—S2—C24	108.8 (3)	H15A—C15—H15B	109.5
O8—S2—C24	102.6 (2)	C12—C15—H15C	109.5
C6—O1—H1A	118 (4)	H15A—C15—H15C	109.5
C3—O4—S1	117.1 (3)	H15B—C15—H15C	109.5
C21—O5—H5	114 (4)	C17—C16—N2	106.2 (4)
C18—O8—S2	117.3 (3)	C17—C16—C22	114.2 (4)
C1—N1—C8	115.1 (4)	N2—C16—C22	101.9 (3)
C1—N1—C5	101.2 (4)	C17—C16—H16	111.4
C8—N1—C5	115.8 (4)	N2—C16—H16	111.4
C1—N1—H1B	103 (3)	C22—C16—H16	111.4
C8—N1—H1B	111 (3)	C18—C17—C16	115.5 (4)
C5—N1—H1B	110 (3)	C18—C17—H17A	108.4
C23—N2—C20	116.0 (3)	C16—C17—H17A	108.4
C23—N2—C16	114.7 (4)	C18—C17—H17B	108.4
C20—N2—C16	101.4 (3)	C16—C17—H17B	108.4
C23—N2—H2	103 (3)	H17A—C17—H17B	107.5
C20—N2—H2	116 (3)	O8—C18—C17	110.0 (4)
C16—N2—H2	105 (3)	O8—C18—C19	106.6 (3)
C2—C1—N1	108.7 (4)	C17—C18—C19	113.1 (4)
C2—C1—C7	115.9 (5)	O8—C18—H18	109.0
N1—C1—C7	101.4 (4)	C17—C18—H18	109.0
C2—C1—H1	110.2	C19—C18—H18	109.0
N1—C1—H1	110.2	C20—C19—C18	113.7 (4)
C7—C1—H1	110.2	C20—C19—H19A	108.8
C1—C2—C3	114.0 (5)	C18—C19—H19A	108.8
C1—C2—H2A	108.7	C20—C19—H19B	108.8
C3—C2—H2A	108.7	C18—C19—H19B	108.8
C1—C2—H2B	108.7	H19A—C19—H19B	107.7
C3—C2—H2B	108.7	N2—C20—C19	105.8 (3)
H2A—C2—H2B	107.6	N2—C20—C21	104.9 (4)
O4—C3—C2	107.5 (4)	C19—C20—C21	113.5 (4)
O4—C3—C4	108.4 (4)	N2—C20—H20	110.8
C2—C3—C4	113.4 (4)	C19—C20—H20	110.8
O4—C3—H3	109.1	C21—C20—H20	110.8
C2—C3—H3	109.1	O5—C21—C20	114.0 (4)
C4—C3—H3	109.1	O5—C21—C22	113.7 (4)
C3—C4—C5	113.2 (3)	C20—C21—C22	103.4 (3)
C3—C4—H4A	108.9	O5—C21—H21	108.5
C5—C4—H4A	108.9	C20—C21—H21	108.5
C3—C4—H4B	108.9	C22—C21—H21	108.5
C5—C4—H4B	108.9	C16—C22—C21	106.8 (4)
H4A—C4—H4B	107.7	C16—C22—H22A	110.4
N1—C5—C4	105.9 (4)	C21—C22—H22A	110.4
N1—C5—C6	104.3 (3)	C16—C22—H22B	110.4
C4—C5—C6	114.0 (4)	C21—C22—H22B	110.4
N1—C5—H5A	110.8	H22A—C22—H22B	108.6
C4—C5—H5A	110.8	N2—C23—H23A	109.5
C6—C5—H5A	110.8	N2—C23—H23B	109.5
O1—C6—C5	115.4 (4)	H23A—C23—H23B	109.5
O1—C6—C7	114.1 (4)	N2—C23—H23C	109.5
C5—C6—C7	103.5 (4)	H23A—C23—H23C	109.5
O1—C6—H6	107.8	H23B—C23—H23C	109.5
C5—C6—H6	107.8	C25—C24—C29	120.1 (5)
C7—C6—H6	107.8	C25—C24—S2	121.5 (4)
C6—C7—C1	105.9 (4)	C29—C24—S2	118.4 (4)
C6—C7—H7A	110.6	C24—C25—C26	118.6 (5)
C1—C7—H7A	110.6	C24—C25—H25	120.7
C6—C7—H7B	110.6	C26—C25—H25	120.7
C1—C7—H7B	110.6	C27—C26—C25	123.1 (5)
H7A—C7—H7B	108.7	C27—C26—H26	118.4
N1—C8—H8A	109.5	C25—C26—H26	118.4
N1—C8—H8B	109.5	C26—C27—C28	117.1 (5)
H8A—C8—H8B	109.5	C26—C27—C30	122.5 (6)
N1—C8—H8C	109.5	C28—C27—C30	120.4 (5)
H8A—C8—H8C	109.5	C29—C28—C27	122.3 (5)
H8B—C8—H8C	109.5	C29—C28—H28	118.8
C10—C9—C14	120.6 (5)	C27—C28—H28	118.8
C10—C9—S1	120.7 (4)	C28—C29—C24	118.7 (6)
C14—C9—S1	118.7 (4)	C28—C29—H29	120.6
C9—C10—C11	120.1 (5)	C24—C29—H29	120.6
C9—C10—H10	119.9	C27—C30—H30A	109.5
C11—C10—H10	119.9	C27—C30—H30B	109.5
C10—C11—C12	120.3 (5)	H30A—C30—H30B	109.5
C10—C11—H11	119.8	C27—C30—H30C	109.5
C12—C11—H11	119.8	H30A—C30—H30C	109.5
C13—C12—C11	117.9 (5)	H30B—C30—H30C	109.5
			
O2—S1—O4—C3	55.1 (4)	C12—C13—C14—C9	−1.0 (9)
O3—S1—O4—C3	−175.8 (3)	C10—C9—C14—C13	0.5 (8)
C9—S1—O4—C3	−60.8 (4)	S1—C9—C14—C13	178.1 (4)
O6—S2—O8—C18	175.5 (3)	C23—N2—C16—C17	159.3 (4)
O7—S2—O8—C18	−55.1 (4)	C20—N2—C16—C17	−74.9 (4)
C24—S2—O8—C18	60.3 (3)	C23—N2—C16—C22	−80.9 (5)
C8—N1—C1—C2	159.1 (4)	C20—N2—C16—C22	44.9 (4)
C5—N1—C1—C2	−75.3 (5)	N2—C16—C17—C18	55.6 (5)
C8—N1—C1—C7	−78.4 (5)	C22—C16—C17—C18	−55.8 (6)
C5—N1—C1—C7	47.2 (4)	S2—O8—C18—C17	108.5 (4)
N1—C1—C2—C3	56.0 (6)	S2—O8—C18—C19	−128.5 (4)
C7—C1—C2—C3	−57.3 (6)	C16—C17—C18—O8	83.9 (5)
S1—O4—C3—C2	122.0 (4)	C16—C17—C18—C19	−35.1 (6)
S1—O4—C3—C4	−115.1 (4)	O8—C18—C19—C20	−85.3 (5)
C1—C2—C3—O4	84.9 (6)	C17—C18—C19—C20	35.7 (6)
C1—C2—C3—C4	−34.9 (6)	C23—N2—C20—C19	−158.9 (4)
O4—C3—C4—C5	−83.1 (5)	C16—N2—C20—C19	76.2 (4)
C2—C3—C4—C5	36.2 (6)	C23—N2—C20—C21	80.8 (5)
C1—N1—C5—C4	75.0 (4)	C16—N2—C20—C21	−44.1 (4)
C8—N1—C5—C4	−159.9 (4)	C18—C19—C20—N2	−57.6 (5)
C1—N1—C5—C6	−45.6 (5)	C18—C19—C20—C21	56.8 (5)
C8—N1—C5—C6	79.6 (5)	N2—C20—C21—O5	−98.7 (4)
C3—C4—C5—N1	−57.4 (5)	C19—C20—C21—O5	146.3 (4)
C3—C4—C5—C6	56.7 (5)	N2—C20—C21—C22	25.2 (5)
N1—C5—C6—O1	−100.7 (4)	C19—C20—C21—C22	−89.8 (5)
C4—C5—C6—O1	144.3 (4)	C17—C16—C22—C21	84.4 (5)
N1—C5—C6—C7	24.6 (5)	N2—C16—C22—C21	−29.6 (5)
C4—C5—C6—C7	−90.4 (4)	O5—C21—C22—C16	127.2 (4)
O1—C6—C7—C1	130.7 (4)	C20—C21—C22—C16	3.1 (5)
C5—C6—C7—C1	4.5 (5)	O6—S2—C24—C25	135.6 (4)
C2—C1—C7—C6	85.2 (5)	O7—S2—C24—C25	1.3 (5)
N1—C1—C7—C6	−32.2 (5)	O8—S2—C24—C25	−114.3 (4)
O2—S1—C9—C10	−5.8 (5)	O6—S2—C24—C29	−44.9 (5)
O3—S1—C9—C10	−137.9 (4)	O7—S2—C24—C29	−179.2 (4)
O4—S1—C9—C10	111.1 (4)	O8—S2—C24—C29	65.2 (4)
O2—S1—C9—C14	176.6 (4)	C29—C24—C25—C26	−0.9 (8)
O3—S1—C9—C14	44.5 (5)	S2—C24—C25—C26	178.6 (4)
O4—S1—C9—C14	−66.5 (4)	C24—C25—C26—C27	−1.4 (8)
C14—C9—C10—C11	−1.0 (8)	C25—C26—C27—C28	2.6 (8)
S1—C9—C10—C11	−178.5 (4)	C25—C26—C27—C30	−178.5 (5)
C9—C10—C11—C12	1.9 (8)	C26—C27—C28—C29	−1.5 (8)
C10—C11—C12—C13	−2.3 (8)	C30—C27—C28—C29	179.5 (6)
C10—C11—C12—C15	177.6 (5)	C27—C28—C29—C24	−0.7 (9)
C11—C12—C13—C14	1.8 (8)	C25—C24—C29—C28	1.9 (8)
C15—C12—C13—C14	−178.0 (5)	S2—C24—C29—C28	−177.6 (4)

### Hydrogen-bond geometry (Å, °)

**Table:**

*D*—H···*A*	*D*—H	H···*A*	*D*···*A*	*D*—H···*A*
O1—H1A···Cl1^i^	0.82 (2)	2.37 (2)	3.185 (4)	170 (6)
O5—H5···Cl2^ii^	0.83 (2)	2.35 (3)	3.156 (4)	164 (6)
N1—H1B···Cl1	0.87 (2)	2.26 (2)	3.091 (4)	159 (4)
N2—H2···Cl2	0.87 (2)	2.19 (2)	3.041 (4)	165 (4)

Symmetry codes: (i) *x*−1, *y*, *z*; (ii) *x*+1, *y*, *z*.
